# The Crosstalk Between Autophagy and MicroRNAs in Esophageal Carcinoma -

**DOI:** 10.31661/gmj.v12i.2903

**Published:** 2023-05-23

**Authors:** Neda Gorjizadeh, Sahar poudineh, Behnaz Barghgir, Mohammadreza Eghbali, Alireza Sarlak, Maryam poudineh

**Affiliations:** ^1^ Department of Internal Medicine, Sina Hospital, Tehran University of Medical Sciences, Tehran, Iran 2 School of Medicine, Mashhad Azad University, Mashhad, Iran; ^2^ School of Medicine, Mashhad Azad University, Mashhad, Iran; ^3^ Student Research Committee, School of Medicine, Shahroud University of Medical Sciences, Shahroud , Iran; ^4^ Student Research Committee, School of Medicine, Hamadan University of Medical Sciences, Hamadan, Iran

**Keywords:** Autophagy, microRNAs, Cancer, Esophageal Carcinoma, Squamous Cell Carcinoma, Therapy

## Abstract

Esophageal cancer (EC) is considered one of the most prevalent and aggressive malignancies worldwide, with a variety of molecular alterations thought to contribute to its incidence, development, progression, and invasion. However, the exact underlying mechanism has not been elucidated. Autophagy is a highly conserved degradative and recycling process that can function with a dual role in either the progression or the treatment of EC. Since microRNAs (miRNAs) are described as upstream regulators capable of controlling both oncogenic pathways and autophagic flux, the present study has aimed to review the crosstalk between autophagy and miRNAs and the potential perspective of these mechanisms in EC prevention and treatment.

## Introduction

Esophageal carcinoma (EC) is considered one of the world’s most prevalent and aggressive types of cancer [1]. Globally, the morbidity and mortality caused by EC are ranked eighth and sixth among all cancer types [2]. Remarkably, the importance of this fetal malignancy has become more significant in particular areas of the world, including Eastern Asia and Southern and Eastern Africa [3]. For instance, it is reported as the fourth leading cause of death among all types of cancer in China [4]. EC is characterized as a complicated catastrophic disorder that can be caused by several different factors in terms of histology and population [5]. Histological shreds of evidence have characterized EC into two major subcategories, including squamous cell carcinoma (ESCC) and adenocarcinoma (EAC) [6]. Anatomically, ESCC predominates in the upper and mid-esophagus, whereas EAC is more prevalent in the lower esophagus. Although significant reductions in behavioral risk factors including alcohol and tobacco consumption have dramatically dropped ESCC incidence rates in the United States of America, gastroesophageal reflux disease and obesity have raised the incidence of EAC in Western countries [7]. Nevertheless, according to statistical data, ESCC remains the cause of approximately 90% of the incidence of EC worldwide [8]. Currently, impressive progress in the manufacture of novel chemotherapeutics with reduced adverse effects [9], as well as extensive research on adjuvant therapies with antitumor properties and increasing drug safety, along with modern diagnostic methods and approved tumor markers, have a beneficial function in the management of chronic diseases, particularly cancer. Consequently, these advances promise to raise overall survival and improve the quality of life for cancer survivors [10]. Despite these tremendous progressions in early diagnosis, novel chemotherapeutic approaches, and surgical techniques, the current outcomes of EC are very poor, as the 5-year survival rate is estimated to be as disappointing as 18%. The poor prognosis of EC has been attributed to lymph node metastasis, diagnosis in advanced stages of the disease, and resistance to current treatment protocols [11]. Therefore, there is a substantial necessity to clarify the molecular mechanisms underlying the growth and progression of EC, to identify non-invasive molecular biomarkers for diagnosing the disease at an early stage and to suggest potential molecular targets to manufacture novel pharmaceutics. The present study aimed to review the crosstalk between microRNAs (miRNAs) and autophagic flux, both in the development and prevention of cancer, after a concise overview of autophagy and miRNAs.

## A Concise Overview of miRNAs

miRNAs are a well-known class of short and non-coding RNAs (an average of 22 nucleotides in length) that have been described as the most important regulators of gene expression. In the early 90s Ambros and Ruvkun groups discovered the first miRNA in Caenorhabditis elegans named lin-4 [12]. However, basic information regarding the biogenesis, function, and mechanism was clarified during the following decades, as well as the identification of subsequent miRNAs; this process is still ongoing, with novel miRNAs being identified and described. Most of the miRNAs are directly transcribed by RNA polymerase II from DNA sequences. The products are known as primary miRNAs, which are transformed into precursor miRNAs and mature miRNAs after processing [13]. Contrary to the traditional assumptions that miRNAs have been described as negative regulators of gene expression, often mediated by the interaction of miRNAs with the 3′ UTR of target mRNAs, coding sequences, and 5′ UTR to suppress expression via mRNA degradation or gene silencing, these short RNAs can induce expression of genes under certain conditions [14]. This less-indicated capability of miRNAs has appeared to be mediated by interactions with gene promoters [15].

## The Biogenesis of and Mechanisms of miRNAs-mediated Control of Gene Expression

The processing of RNA polymerase II/III transcripts is the initial step in miRNA biogenesis. Based on the processing source, miRNAs can be divided into two main categories, intragenic and intergenic [16]. Approximately 50% of the currently discovered miRNAs are classified as intragenic miRNAs, which are often processed from introns and a relatively small number of exons of genes capable of coding proteins. Intergenic miRNAs are independently transcripted of host genes and regulated by their exclusive promoter [16]. miRNAs biogenesis could be accomplished through two distinct pathways, canonical and non-canonical [17].

Until now, different mechanisms have been considered for miRNA-regulated gene expression, including miRNA-mediated gene silencing via the minimal miRNA-induced silencing complex (miRISC) [18], the intranuclear miRNA-mediated transcriptional and posttranscriptional gene expression [19], and miRNA-mediated translational activation [20]. Regardless of the different mechanisms by which miRNAs regulate gene expression, these RNAs are critical for proper cell development, proliferation, and differentiation and are involved in many vital biological processes. Therefore, the aberrant expression of miRNAs has been attributed to various diseases, including cancer. Moreover, the secretion of miRNAs into extracellular fluids, being extensively documented as promising biomarkers of a variety of disorders, is another important perspective of the beneficial function of miRNAs. Furthermore, miRNAs appeared to contribute to cell-cell communications as signaling molecules [21].

## MiRNAs Represent A Prominent Role in Cancer

Interestingly, the first evidence for the role of miRNAs in human diseases and disorders was reported in cancer cells, where Croce et al. demonstrated the frequent deletion of miR-15a/16-1 cluster in chronic lymphocytic leukemia implicated the tumor suppressor function of this miRNA [22]. This report was followed by several studies to clarify the consequences of overexpression or downregulation of miRNAs in various biological disorders. Many of these studies have attributed the regulatory role of miRNAs in cancer progression or suppression to the control of essential biological processes such as autophagy [23]. However, such a role has not been comprehensively elucidated, particularly in EC. The present study attempt to describe the miRNA-mediated regulation of this pivotal process and its association with EC progression/suppression.

## Autophagy Overview

Autophagy, composed of two Greek words auto (self) and phagein (to eat), is a highly conserved catabolic process important for maintaining cellular homeostasis. This process was first described in the 1960s; however, it took nearly 30 years for the exploration of the involved genes, known as autophagy-related (ATG) genes, to make a fundamental breakthrough in construing the mechanistic intricate of autophagy [24]. Several documents divide autophagy into three main types, including macroautophagy, microautophagy, and chaperone-mediated autophagy, all of which are involved in delivering specific cargo to the lysosomes regardless of their different pathways [25]. Initially, autophagy, particularly macroautophagy, was considered a non-selective bulk degrative process. However, it is currently represented as a highly selective process after discovering autophagy receptors, and p62/sequestosome 1 (SQSTM1) was the first [26]. The propound of a specifically selected cargo became the foundation for more division of autophagy, including nucleophagy (parts of the nucleus), lysophagy (lysosomes), ER-phagy (endoplasmic reticulum), mitophagy (mitochondria), glycophagy and lipophagy (macromolecules), and xenophagy (pathogens) [26].

Although autophagy has traditionally been described as a degradative process, it is more appropriate to consider it as a recycling pathway to more precisely represent the physiological function of autophagy. This description originates from the fact that the end products of the autophagy pathway are not disappeared but are used as an energy source or as a building block for the biosynthesis of other macromolecules. Importantly, a plethora of documents demonstrated that this process considerably participates in a variety of pivotal physiological processes ranging from starvation to adaptation, damaged or excessive organelles turnover, aberrant structures degradation, innate and adaptive immunity, cell development and differentiation, tumor suppression, cell survival and programmed cell death [27]. As a result, the beneficial role of autophagic flux is attributed to different aspects of human and other organisms’ physiology and pathology, including metabolism and energy homeostasis, cell development and proliferation, differentiation, and death [28].

## Autophagic Flux 

Macroautophagy, has been described as the most important and best studied type of autophagy, generally consisted of two stages, including the generation of specific structures known as autophagosome and the delivery of selected cargo to the lysosome. It is quite clear that such a complex and highly conserved process must be controlled by upstream regulatory mechanisms. 

In addition to all the regulatory mechanisms, miRNAs widely regulate the autophagic flux. Regulation of autophagy by miRNAs can be described in two levels, including regulation of upstream signaling pathways (e.g. mTOR) and regulation of autophagic flux (e.g. initiation, phagophore nucleation, elongation, autophagosome maturation, and autolysosome formation) [23]. Recently, there has been a strong interest in considering miRNA-mediated regulation of autophagy as a promising cancer therapy strategy due to the wide range of potential targets [29]. However, it is highly necessary to determine whether the role of autophagy in cancer is preventive or accelerative.

## Autophagy in Cancer

The role of autophagy in cancer can be considered contradictory as it prevents the transformation of normal cells into malignant types; however, once cancer is established, it provides the possibility of survival and growth of tumor cells [30]. Early reports of monoallelic loss of the beclin 1 (BECN1) encoding gene in 40 to 75% of breast, prostate, and ovarian cancers led to describing autophagy as a tumor suppressor [31]. Further studies found that suppression of autophagy was associated with the growth of cancer cells, and BECN1 heterozygous mutant animals were prone to the progress of liver and lung tumors and lymphomas [32]. However, it is reported that the allelic loss of BECN1 is followed by the promotion of p53 activation and, thereby reduction in tumorogenesis, challenging the belief in the tumor suppressor role of the BECN1 and consequently autophagy [33]. The determination of the induction of benign hepatocarcinoma due to autophagy deficiency in mice, although confirming the tumor suppressive role of this highly conserved process, suggests that autophagy is necessary for tumor progression and transforming to malignancy [34].

Complicatedly, cancer cells seem to rely on autophagy more than normal cells and tissues due to the inherent deficiencies in the microenvironment of tumor cells, in addition to increased demands for energy, metabolism, and biosynthesis inflicted by dysregulated excessive proliferation of tumor cells [35]. The upregulation of autophagy in RAS-transformed tumor cells leads to enhances development, survival, tumorigenesis, and metastasis [36]. Interestingly, the dependence of RAS-driven cancers on autophagy is as high as they are known as "autophagy addicted" [37]. Furthermore, it is documented that autophagy is crucially required for carcinoma fate in non-small cell lung cancer. Desirably, the defect in autophagy or inactivation of autophagic flux is related to the genesis of human oncocytomas and the progression of lung carcinomas to benign disease suggesting the promising therapeutic function of autophagy [38].

## The Crosstalk Between miRNAs and Autophagy in EC 

Considering the regulatory role that miRNAs play on pivotal cellular processes such as growth, development, proliferation, differentiation, and regulated cell death pathways (e.g. apoptosis and autophagy), their dysregulation in EC is not surprising [39][40]. This dysregulation in the expression of miRNAs may promote tumor suppression or promote tumor progression. miR-1224-5p, for example, can directly target tensin 4 (TNS4) gene, decrease the levels of tensin 4 protein, and thereby inactivate the EGFR-EFNA1/EPHA2-VEGFA signaling pathway, all of which lead to the suppression of proliferation, migration, and invasion of ESCC cells [41]. Otherwise, miR-145 is effective in ESCC growth, proliferation, and invasion by directly targeting SMAD5 and it is considered a biomarker of late-stage ESCC, poor prognosis, unfavorable therapeutic outcomes, and a potential target for both diagnostic tools and therapeutic strategies [42].

Similarly, ECs are associated with profound changes in autophagy-related gene- and protein-level expression. In addition to the tumor-suppressive role of autophagy, it has been reported that the expression of autophagy genes is significantly reduced in human ESCC lesions [43]. Deep deletions in ATG7, whose product contributes to autophagosome elongation, and as a result, lower levels of ATG 7 and autophagic flux have been reported in ESCC [44]. Similarly, the reduction and/or lost expression of BECN1, being considered the effector of autophagosome nucleation, is demonstrated in ESCC [45]. Subsequent studies revealed that ATG7 closely associates with the NFE2L2, whose product is a mediator of antioxidant processes known as NRF2; hence it can be assumed that ATG7 represents a preventive antitumor function [46]. BECN1 is involved in confronting the growth, progression, and invasion of established ESCC cells [47]. In addition, aberrant methylation of transcriptional variant 1 of the LC3A isoform, known as LC3Av1, concurrent with disrupted protein levels, has been identified in human ESCC tumors [48]. As autophagy has been demonstrated to have a dual role in promoting cell survival and programmed cell death, it is a reasonable expectation to consider the possibility of increased ATG levels in EC lesions. For example, several studies have reported elevated levels of LC3 [49]. In fact, the levels of LC3 gradually increased during the progression from premalignant lesions to early ESCC, which exhibited a positive correlation with a proliferation marker named Ki-67. Although this increase in the level of LC3 was identified in the early stages, in the late stages, unchanged levels were reported [50]. Moreover, the upregulation of ATG5, a mediator of autophagosome elongation, and ULK1, a kinase involved in the phosphorylation of BECN1 and initiation of autophagy, has been identified in ESCC lesions [51]. Furthermore, elevated levels of a marker of mitophagy, PTEN-induced putative kinase 1 (PINK), suggest the upregulation of mitophagy in human ESCC lesions [49].

In EC cells, the contradictory alteration in the expression of ATG genes, regulation of autophagy gene by miRNAs, and dysregulation of miRNAs suggest an unelucidated crosstalk between miRNAs and autophagy that may play a role in preventing and countering proliferation and invasion of established cells may play a role in growth and development and malignancy. Considering the dual role of autophagy and the tumor-suppressive or oncogenic functions of miRNAs, it is logical to expect such crosstalk would be involved in confronting growth, development, and metastasis or contribute to the promotion of EC progression and invasion. Therefore, tumor suppression or tumor promotion are two major perspectives that can describe the role of such crosstalk in EC [52]. 

An integrated framework study on ESCC cells revealed that autophagy is regulated by three different death-related miRNAs, including miR-20b, miR-196, and miR-498 [53]. In the hypoxic ESCC cells microenvironment, the altered expression of miRNAs leads to the alteration in several signaling pathways in which autophagic flux is mentioned as one of the key pathways [54]. It is reported that miR-1299 is able to modulate the function of the Akt-mTOR pathway via directly bind to the 3′-untranslated region of the upstream regulator, epidermal growth factor receptor (EGFR), thereby promoting the autophagic flux leading to inhibition of ESCC progression [55]. Similarly, miR‑144 directly targets TP53‑inducible glycolysis and apoptosis regulator (TIGAR) and activates autophagic flux, all of which results in the inhibition of ESCC progression, colony formation, and invasion, and promotion of tumor cell death [56]. Furthermore, miR-382 is capable of suppressing ESCC proliferation and colony formation as well as promoting the arrest of the cell cycle at the G2/M phase via the induction of apoptosis and autophagy [57].

As expected, previous studies have documented that the interaction between miRNAs and ATGs is not limited to the suppression of EC progression but also significantly contributes to promoting cancer proliferation and invasion. For instance, miR-126 could downregulate STAT3 levels by directly binds to the 3′-untranslated region. STAT3 is considered the regulator of apoptosis-related genes (e.g. Bcl-xL, Bcl-2, and VEGF) and the upstream controllers of autophagic flux (BECN1, HIF1A, AMPK, PI3K, and miRNAs) [58][59]. Thereby, targeting STAT3 by miR-126 results in the inhibition of both autophagy and apoptosis in ESCC, which is followed by the development of tumorigenesis [60]. Whereas, the activation of the cell death programs caused by miRNAs, such as miR-634, may be followed by desired effects including augmentation of cytotoxicity after chemotherapy in ESCC cells [61]. Conversely, it has been reported that miR-638, an oncogene capable of promoting cell proliferation, migration, and invasion, contributes to the progression of ESCC malignancy phenotypes via enhancing autophagy through the targeting of DACT3, a key regulator of Wnt/β-catenin signaling [62]. Moreover, the promotion of autophagy by miR-10b, which is mediated by the diminish of a tumor suppressor named DAZAP1, leads to the progression of ESCC [63]. 

The review of these studies has the benefit of successfully suggesting novel diagnostic and therapeutic strategies resulting in the management of the disease. Indeed, several studies have suggested potential diagnostic and therapeutic approaches by considering the crosstalk discussed in the present study. Specifically, Chen *et al*. suggested that in ESCC cells, the inhibition of autophagy by miR-450a-5p increases radiosensitivity, which promises a novel therapeutic approach to confront radioresistant types of the disease [64]. In addition, well-known chemicals such as galectin-9, a tandem-repeat type galectin with anti-proliferative properties against tumor cell types, and polygalactin D, a bioactive compound representing antitumor properties, have shown benefits in ESCC treatment by inducing alterations in the expression of miRNAs and ATGs [65][66]. However, the conducted studies appear to be insufficient, and further studies followed by extensive clinical trials are crucially required.

## Conclusion

miRNAs and autophagy are two key regulators of cell proliferation and differentiation, which are misregulated during the cellular transformation from normal cells to malignant types. 

Dysregulation in the expression of both miRNAs and autophagy represents contradictory outcomes as both upregulation and downregulation in each of them may be followed by undesired consequences. More importantly, the interaction of miRNAs and ATGs has been reported in both EC suppression and progression, which can suggest promising diagnostic, prognostic, and therapeutic approaches. However, in order to achieve this objective, further studies are encouraged.

## Conflicts of Interest

There are no conflicts of interest to declare.
